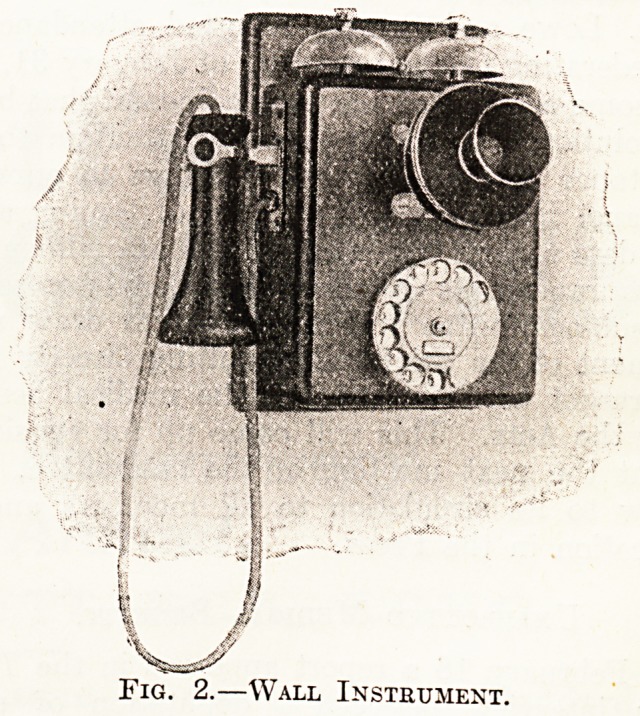# Automatic Telephones for Institutions

**Published:** 1914-02-28

**Authors:** 


### AUTOMATIC TELEPHONES FOR INSTITUTIONS.

### The Composite Installation at New "King's."

With the increase in size of our modern hospitals the question of internal communication is one which must necessarily engage the close attention of the managers of every institution which maintains a high standard of general efficiency. The old-fashioned speaking-tube, always insanitary, and, more often than not, ineffective; various systems of internal telephones, of which the ordinary inter-communication telephone, for which no operator is required, is probably best, but to extend which is an expensive undertaking; and the manual telephone apparatus have all been tried, but none of them have proved adequate or economically efficient. Of late electricians have turned their attention to the difficult problem o finternal communication, and Messrs. Siemens Bros. and Co., of Woolwich, have installed a system which

Fig. 1.—Table Instrument.

has now been in constant operation for six months at King's College Hospital, Denmark Hill, which has proved satisfactory. It consists of a complete automatic telephone system which includes, in addition, an equipment of electric clocks, staff arrival indicators, and miscellaneous bells, all worked by the same battery of accumulators as is used for the telephones. The whole installation is auto-controlled (even the clocks being self-winding), and requires no attention beyond an occasional inspection.

### The Automatic Telephone System Briefly Explained.

Space will not permit of a detailed description, but we will endeavour to give a brief outline of the system in order that our readers may form some idea of the advantages pertaining to automatic telephony.

The receiving and transmitting instrument is of the same pattern as that employed in the ordinary manual service, with the addition of a small dial fitted to the base. In the dial there are ten finger-holes, below which appear numbers 0 to 1 (see figs. 1 and 2).

To make a call the receiver is first removed from off the rest; a finger is inserted in one of the numbered holes, and the dial is pulled round as far as it will go. The finger is then removed and the dial returns to its normal position. This action is repeated for each digit contained in the number of the subscriber required. As soon as the last digit has been "pulled" in this way connection is automatically effected, and if the person called is disengaged his instrument bell is immediately rung for one second, and continues to ring at intervals of five seconds until he answers. During the time the bell is ringing the calling subscriber receives a high-toned note in his receiver, which indicates that the distant bell is ringing. Should, however, the called subscriber be already engaged a burring note is received immediately after the last digit is sent, so that the calling subscriber is instantly made aware of the fact that connection at that moment is impossible. Directly the receiver is hung up the whole apparatus is restored to normal, and the instrument is free to make or receive further calls.

It will be seen from these few remarks that with accuracy and speed of connection the chief defects of the manual system are practically eliminated, and since it is absolutely impossible for any conversation to be "tapped," it is obvious that automatic telephony provides an ideal means, not only of internal, but of general communication, and it is unquestionably a system with a future.

### Cost of an Installation and Maintenance.

The cost of an installation must necessarily differ with varying conditions. For small equipments, however, the initial cost of an automatic is greater than that of a manual system of equal size, but apart altogether from the many advantages of an automatic working, it has been proved, from observations existing over a number of years, to be the most economical from a comparison of the annual charges for maintenance. The interest, depre-

Fig. 2.—Wall Instrument.

ciation, and maintenance on a small manual equipment equals approximately 14 per cent. per annum of the capital sum expended, and this figure is also applicable to an automatic plant of equal size, but in the case of the manual equipment the operating cost must be added, including the necessary relief operators for meal-times, illness, and holidays, whilst further allowance must be made if a continuous day and night service is required.

The question of space occupied should also be taken into consideration. For a manual switchboard a comparatively light, airy room must be provided, in which the operators work, whereas the automatic equipment needs no attention beyond an occasional inspection, takes up very little space, and can be placed in any available position beside a wall, or even in the basement, provided, of course, the place selected is reasonably dry.

## Figures and Tables

**Fig. 1. f1:**
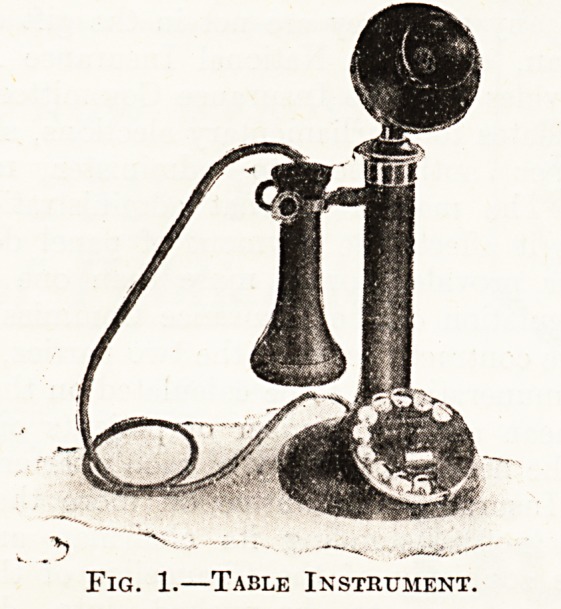


**Fig. 2. f2:**